# A Mental Health Liaison Core-24 Quality Improvement Project (QIP): Improving Recording of Causes of One-Hour Target Breaches of Referrals to a Mental Health Liaison Team by an A&E Department

**DOI:** 10.1192/bjo.2026.11362

**Published:** 2026-06-30

**Authors:** Aoife Clark-McGhee

**Affiliations:** Sussex Partnership NHS Foundation Trust, Brighton & Hove, United Kingdom

## Abstract

**Aims:**

Primary: To increase the proportion of A&E referrals to the Mental Health Liaison Team (MHLT) at a medium-sized district general hospital with documented reasons for breaching the one-hour response target. Secondary: To improve the team's shared understanding of breach causes.

**Methods:**

The MHLT 'A&E Assessment' proforma separately records referral time and assessment initiation time from which monthly ‘breach’ statistics are produced. November 2025 data was audited to establish whether ‘breach reasons’ were consistently recorded. During early November open-forum team consultations occurred where frequent breach codes were agreed, these were: 1: Multiple referrals within 1 hour, 2: Staffing levels–shortages/sickness, 3: Patient not medically fit, 4: Patient left department, 5: Other, specify. The proforma was amended accordingly and was emailed to all team members on 17.12.2025. All were advised of the audit and encouraged to use the new proforma, with posters in the MHLT office acting as a further reminder. Data was re-audited between 18.12.25–17.1.26 to determine overall breaches (with reason recorded) and percentages per category.

**Results:**

In November 2025 (30 days) MHLT accepted 97 A&E referrals. 32 breached the one-hour target (33%), 10 recorded a breach reason (30%).

From 18 December 2025–17 January 2026 (31 days) MHLT accepted 44 A&E referrals. 20 referrals breached the target (45.5%), 9 recorded reasons (45%) as follows: Code1: 1 (11%), Code2: 6 (67%), Code3: 0 (0%), Code4: 1 (11%), Code5: 1 (11%).

**Conclusion:**

The re-audit showed a 50% increase in recording of breach causes. Team agreement on common breach reasons supported engagement, and the project showed that embedded prompts can improve recording. Documentation rates remain low however, reflecting ongoing service pressures (see Code2 results). It is suggested that further reinforcement through amended team systems will support improved documentation e.g. staff induction, education and embedded prompts. Notably 50% fewer referrals were accepted during the re-audit phase. An influencing factor might be audit timing (Christmas/New Year). Re-auditing over a longer period is advised whilst cross-referencing against staffing levels.

